# Usability of a new digital walking program for older adults: a pilot study

**DOI:** 10.1186/s12877-023-03739-y

**Published:** 2023-04-01

**Authors:** Jisan Lee, Hyeongju Ryu

**Affiliations:** 1 Department of Nursing, Gangneung-Wonju National University, Wonju, Republic of Korea; 2 Department of Nursing, Konkuk University, Chungju, Republic of Korea

**Keywords:** Social Networking, Wearable Electronic Devices, Gamification, Social Media, Health Promotion

## Abstract

**Background:**

Walking has been emphasized as an important solution for preventing isolation among older adults, especially given the coronavirus disease 2019 (COVID-19) pandemic, and various methods are being proposed to promote walking in this population. In this study, a walking exercise program for the elderly was developed using various latest technologies, and the effectiveness and influencing factors of the program were evaluated.

**Method:**

The walking program for older adults was designed using mHealth devices, social media application, and gamified elements to prevent isolation. Twelve participants were recruited via an online community of retired individuals. This one-year study involved a one-group repeated measures experimental design; an online questionnaire was conducted four times along with a focus group interview.

**Result:**

The results showed that the e-health literacy of the 12 participants increased, while Wearable Device App Literacy/Usability, digital health self-efficacy, and walking program evaluation showed a decline. In the focus group interview, participants expressed their appreciation for this program, ways to overcome its limitations, and expectations for the next program.

**Conclusion:**

This study confirmed the positive effect of the online walking program for retired older adults, indicating that an online-offline convergence program suitable for the “COVID-19 era” will be necessary in the future.

## Background

The world is in great chaos due to the ongoing coronavirus disease 2019 (COVID-19) pandemic [1]. COVID-19 is clinically characterized by fever, cough, fatigue, shortness of breath, pneumonia and other respiratory symptoms, renal failure, neurological symptoms, and even fatal outcomes primarily in individuals with pre-existing disease and in geriatric patients [2–4].

Social distancing has emerged as a necessity [5] to prevent infection and has become a major strategy for dealing with COVID-19 in many countries worldwide [6]. Social distancing is an effective way to prevent the spread of the virus, but the resulting physical activity restriction has led to population isolation, which is a serious concern, especially for older adults, as they are at high risk of not only physical but also mental health problems [7]. The impact of the global pandemic on the older adult population shows that they require a high level of monitoring and care [8]. In other words, solutions to the problems of isolation and health care for older adults due to COVID-19 are warranted.

Exercise has been suggested as an important measure to prevent depression caused by social isolation and to boost immunity during the COVID-19 pandemic. Several studies have highlighted the positive effects of exercise during a pandemic [9]. Proper exercise not only helps with health management, but has also been found to positively affect influenza vaccination response and prolong antibody levels in older adults [10]. Unlike treatments that provide quick results, such as drugs, it is important to exercise regularly to achieve great results [11]. In addition, an active lifestyle may boost immunity, delay aging [12], and reduce the risk of infection in older adults [13].

As a means to solve these problems, digital medicine has recently come in the spotlight. Digital health includes mHealth; this includes mHealth apps (smartphone applications related to health), mHealth devices (wearable devices such as smart arm band, Bluetooth body scale), and personalized medicines [14]. In particular, mHealth apps and devices promote behavior change by adding coaching or incentives to smart wearable devices [15]. Smart wearable devices could play an important role in monitoring and tracking the health of older adults besides providing other benefits for their health [16]. Wearable wrist devices have a significant effect on exercise management and motivation of older adults [17]. Some studies conducted with older adults walking and using wearable devices have confirmed significant physical effects such as increase in physical activity, decrease in body mass index and waist circumference, and decrease in blood pressure [18–23]. As indicated above, several studies have conducted wearable device-based exercise programs for the elderly and compared the physical, physiological, and psychological differences in them before and after the program.

Previous studies have verified the effectiveness of community-centered walking exercise programs, but none have considered using wearable devices with older adults and online communities such as social media. In addition, it was difficult to find studies focusing on the difficulties of older adults in using devices or on their level of digital literacy.

Thus, the aim of this study was to evaluate a walking exercise program for older adults using wearable devices and social media, and to identify factors affecting the program satisfaction.

## Method

### Recruitment of participants

This study was conducted from October 7, 2020 to October 26, 2021. Participants were recruited by sending emails about the program to an online community. Participants were selected based on the following criteria: (1) adults over 60 years old who use smartphones in their daily life; (2) physical ability to participate in a walking program; (3) a personal belief that they are sufficiently capable of participating in the walking program. Those who did not meet the study’s criteria were excluded.

Data were collected after deliberation and approval by the Institutional Bioethics Review Committee for ethical protection of study participants (IRB No.: 1041231–201,110-HR-117–02). The data collection period was from October 7, 2020 to October 26, 2021, and the purpose of the study, program contents, and methods were explained when conducting the preliminary survey. Consent was taken before participation.

### Walking program design

**Fig. 1:**
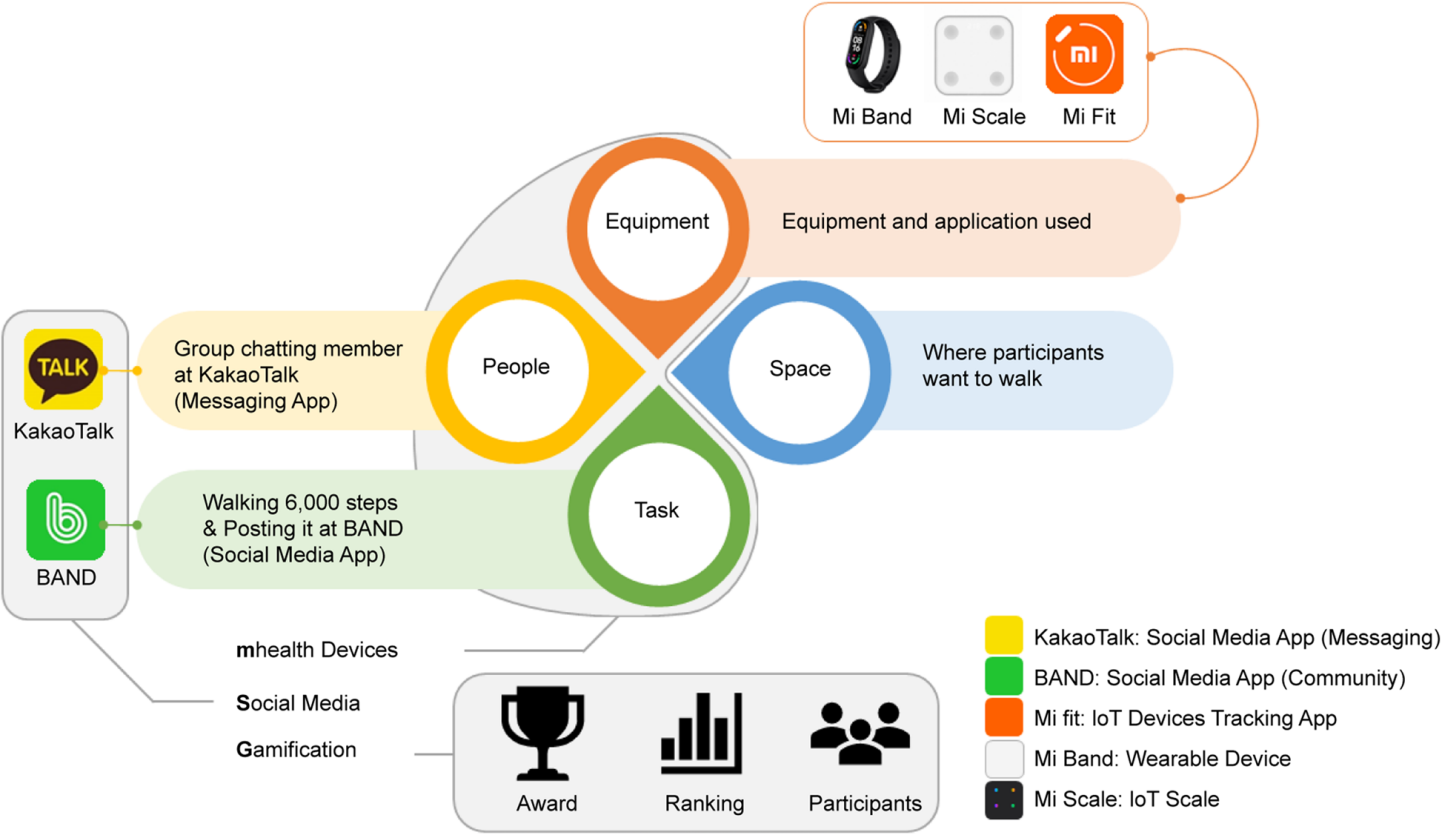
Program construction (based on the STEP model)

Gamified technology for older adults benefits them physically, cognitively, socially, and emotionally [24]; the motive of the game is socializing and they prefer collaboration rather than competition [25]. In order to execute a walking program for older adults during COVID-19, it had to be completely online. To satisfy all participants with different average steps, the walking exercise program was constructed using the STEP model. The STEP model is a widely used framework in the field of physical education, one that composes a relatively simple exercise program. The model comprises four main activity components – space, task, equipment, and people – that can be modified to meet the needs of each individual participant and provide a supportive learning environment [26]. Therefore, in this study, a gamified walking program, called the MSG walking program (**m**Health devices and **S**ocial media apps using **G**amified walking program), was designed to enable socializing in conjunction with mHealth apps and devices to operate an online walking program for retired older adults during COVID-19 as shown in Fig. 1.

The detailed composition of the MSG walking program based on the STEP model is as follows.

• Space: Participants were allowed to walk anywhere they wanted. The degree of freedom of MSG walking program was high since time and place restrictions were not placed on the participants.
• Task: The BAND app (NAVER Corp., Korea), which is the most commonly used social media application by people over 50 years old living in Korea [27], was used to authenticate the target number of steps and allowed participants to encourage and communicate with each other. After the researcher explained the importance of walking in the first online meeting and the participants discussed it together, the daily step goal was set as 6000 steps in consideration of the health advantages [28]. The BAND app was used to post the step count and communicate and motivate each other using comments and likes. The number of steps was verified through the screen capture function of the Mi Band application. The mini-award, which was held monthly for five months from the start of the study, was conducted through the social media app.
• Equipment: Wearable devices that can count steps (Mi Smart Band 5, Xiaomi Inc, China) and the IoT Scale (Mi Body Composition Scale, Xiaomi Inc, China) were utilized. The user’s measurement data collected by both devices can be viewed on the Mi Fit (Anhui Huami Information Technology Co., Ltd., China) app. Accordingly, participants were provided with instructions on how to use the Mi Fit app.
• People: The KakaoTalk app (Kakao Corp., Korea) was used among Messaging Apps for group chatting where all participants communicated with and encouraged each other. Unlike the BAND app, the KakaoTalk app was used to deliver announcements or for real time communication between participants. KakaoTalk is the most popular mobile messaging app in Korea, used by about 97.5% of smartphone users in 2020 [29, 30]. It was used for posting announcements essential for research participation, such as links to online video conferencing for program participation, training, and online meetings, and links to online surveys. Research participation education (instructions on how to use wearable devices, social media applications, and Bluetooth body scale) was conducted in the form of video or interactive news and shared as video clips or images.

### Questionnaire

In this study, various digital health factors and devices such as wearable devices and smart phones were used to enhance participation in the program. To evaluate the change of knowledge and beliefs of the participants related to the effectiveness of this program and device use, an online survey was conducted at four timepoints: Pre, Mid 1 (1.4 month), Mid 2 (5.1 month), and Post (10.3 month).

The composition of the questionnaire is shown in Table 1. A higher score indicated higher satisfaction or a positive result for an item measurement, and a lower score indicated a negative result.

**Table 1:** Composition of the questionnaire

Categories	Timepoint	Sub-categories	Item description	Scale	Reliability (Cronbach’s α)	Source
General characteristic	• Pre • Mid 1 • Mid 2 • Post	• General characteristics (five items) • Health related characteristics (eight items)	• Sex, age, education level, family type, Height and weight, diagnosed disease(s), self-conceived health status	N/A^a^		
Digital health related characteristic	• Pre	• Digital health related characteristic (four items)	• Frequency of contact with health information, search methods, Internet use time, Internet use purpose	N/A^a^		
Cognitive age	• Post	• Four items	• One item each for four age dimensions (feel-age, look-age, do-age, and interest-age)	Category (20 s to 80 s)	-	Barak & Schiffman, 1981
E-Health Literacy	• Pre • Mid 1 • Mid 2 • Post	• Eight items	• Self-report measures an individual's ability to search, use, compare and evaluate health information	Likert scale ranging from 1 to 5	.88	Park, 2017
Wearable Device App Literacy	• Pre • Mid 1 • Mid 2 • Post	• Nine items	• Quiz on interpreting application screenshot	0 to 1	.82	Kim, 2008
Wearable Device App Usability	• Pre • Mid 1 • Mid 2 • Post	• 20 items	• Effect (three items), Perceived Ease (five items), Perceived Usability (nine items), and User Control (three items)	Likert scale ranging from 1 to 5	.85-.92	Schnall, Cho & Liu, 2018
Digital health self-efficacy	• Pre • Mid 1 • Mid 2 • Post	• 20 items	• Self-report questions related to terminology, problem solving, installation and utilization	Likert scale ranging from 1 to 5	.85-.90	Kim, 2017
Usefulness of Body Composition Scale	• Post	• 4 items	• Difficulty connecting to mobile • Usefulness of the scale	Likert scale ranging from 1 to 4		
MSG walking program evaluation	• Pre • Mid 1 • Mid 2 • Post	• Nine items	• Program satisfaction, recommendation, suitability of application used • Program usage fee (one open-ended question)	Likert scale ranging from 1 to 5	-	-

^a^N/A: not applicable

#### General characteristics

It consisted of 17 items on the socioeconomic and health status of the participants.

#### Digital health related characteristics

In order to measure the participants' ability of and interest in using the Internet for health information, multiple-choice questions on frequency of contact with health information, search methods, and Internet usage time and purpose were constructed.

#### Cognitive age

For cognitive age, a tool measuring the four dimensions (Feel, Look, Do, Interest) of Barak & Schiffman (1981) was used with one item for each dimension. Each age group was measured as a categorical variable [31]. Cognitive age was measured only during post evaluation.

#### E-health literacy

E-health literacy refers to the ability to find, understand, and evaluate health information on the Internet, or to apply and transmit the acquired knowledge to solve health problems [32]. In this study, with reference to existing research tools, the tools modified and supplemented by Park (2017) were used [33, 34].

#### Wearable device app literacy

In order to know how well participants understand and use the app that displays measured information in conjunction with the wearable device, an existing health application literacy tool was utilized, composed of nine quiz questions related to the application [35, 36]. The questions were made using screen captures of the Mi Band application.

#### Wearable device app usability

An existing tool consisting of 20 items was used to evaluate the usability of the applications used in the study. It consists of items on effect, perceived ease, perceived usefulness, and user control. Usability is calculated as the average of those variables. In this study, questionnaire was partially modified to measure the usability of the MI Band app. In a previous study, the Cronbach’s alpha values of the detailed items of the tool ranged from 0.85–0.92 [37].

#### Digital health self-efficacy

To measure smartphone self-efficacy, a tool created by Kim (2017) by supplementing the concept of digital media literacy was modified to fit this study and used to measure digital health self-efficacy [38, 39].

#### Usefulness of body composition scale

This scale measures body fat and weight and transmits the measurement results to the Mi app via Bluetooth. The scale served as a reward for participating in the study till the end without failing. It consists of four newly written, brief questions for this study, about the usefulness and difficulty of using the IoT scale provided as a reward for participation.

#### MSG walking program evaluation

In order to measure the overall satisfaction of this walking exercise program, 9 items were newly composed to be rated on a 5-point Likert scale.

The Questions were about the appropriateness of the program, the needs of the community, the need for wearable devices, and the intention to continue using it.

### Focus group interview

Focus group interview (FGI) was performed two months after the post-intervention evaluation and 12 months after the start of the program. Due to COVID-19, an online interview was conducted through Zoom. The FGI was conducted as a semi-structured interview inquiring about online questionnaire items, the impact of social network use on the program, and the program composition.

### Process of the MSG walking program

**Fig. 2:**
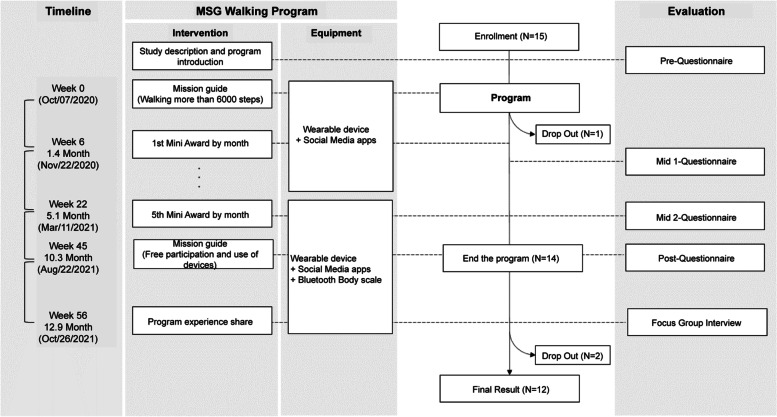
Program process

The MSG walking program was conducted as shown in Fig. 2.

Participants were educated through an online meeting on how to use the mHealth app and other devices to participate in the walking program; a pre-intervention evaluation was also conducted. Participants set their own walking course and walked 6,000 steps every day and disclosed the results to the participant community through the social media app. To prove the number of steps, participants captured the screen of the Mi fit app and uploaded it to the BAND app every day.

The results of the participants' social media activities were scored and the rankings were announced every month; a mini-award was provided as a compliment to the participants with the seven highest scores. The score was calculated by summarizing each user's level of participation, based on the number of posts that achieved the goal and the number of comments or likes on other users' posts.

At the end of the program, an IoT scale was provided as a reward to the participants who participated until the end.

**Fig. 3:**
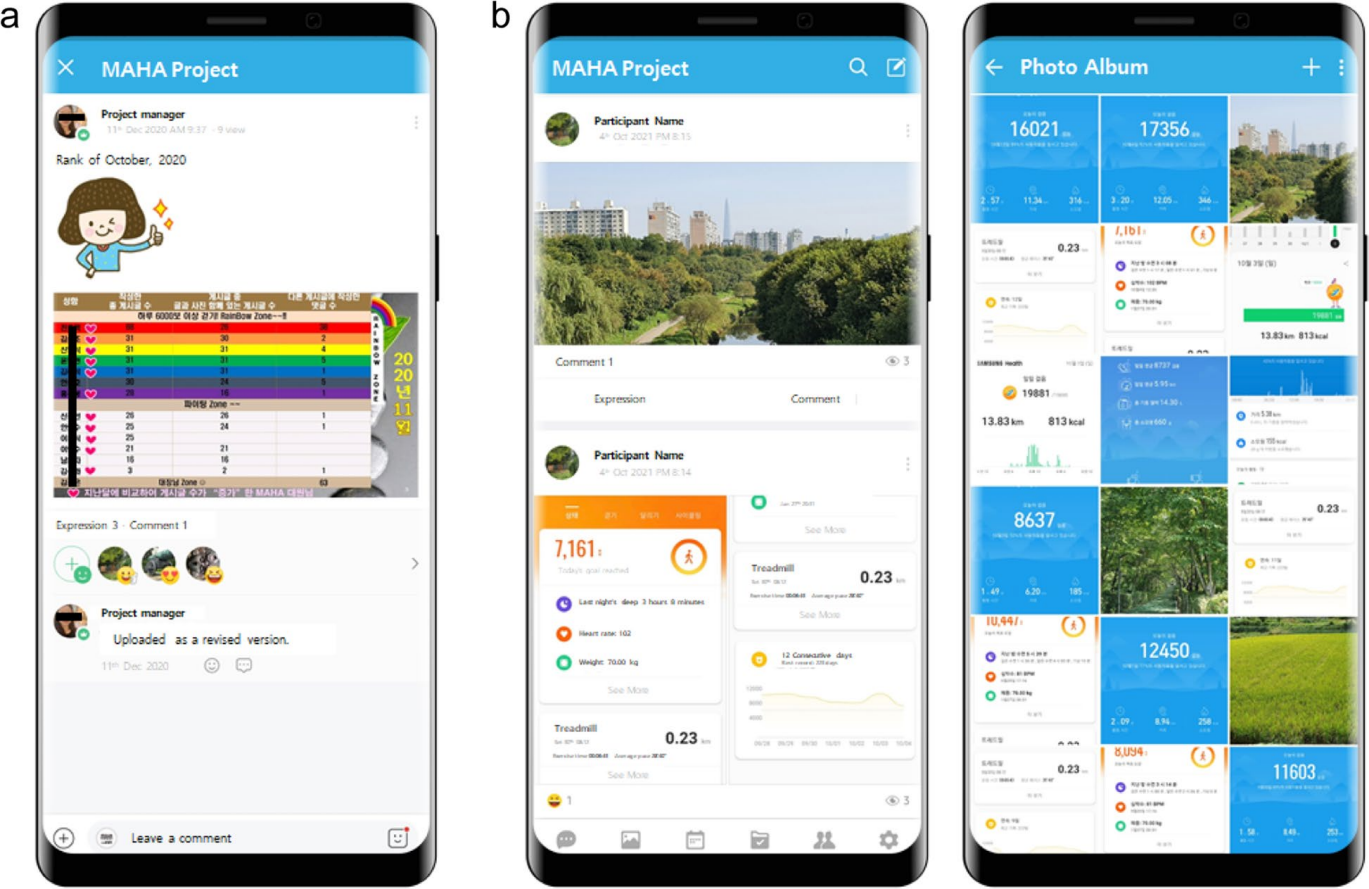
(**a**) Screenshots of Mini Award / (**b**) Screenshots of step count verification uploaded by participants and recommended walking course share

The monthly mini-award was conducted as shown in Fig. 3(a), and the ranking was announced by combining the participation rate. As shown in Fig. 3(b), participants shared their impressions about the day of the walking course as well as the results of their goal achievement on social media, and freely responded to each other's posts.

For data collection, as described previously in Fig. 2, a total of four online surveys and one interview were conducted as follows. The survey was conducted online four times: pre-intervention evaluation before the start of the program; Mid 1 and Mid 2 evaluations 1 month and 5 months into the program period, respectively; and the post-intervention evaluation 10 months after the start of the program. Then, an online FGI was conducted at 12 months.

### Data analysis

The effects of usability, e-health literacy, and exercise programs, excluding app literacy, were measured on a Likert scale ranging from 1 to 5. App literacy comprising nine quiz questions with responses on a scale of 0 to 9 was also converted to a five-point scale for easy comparison.

The significance of the differences in measured values before and after the start of the MSG program was tested through Wilcoxon signed rank test.

## Results

Initially, 15 individuals consented to participate in the study. One person left owing to health reasons before the start of the study and data for two participants with a high non-response rate were excluded during analysis. Data from 12 participants were finally used for analysis. Participant characteristics are shown in Table 2.

**Table 2:** Participant characteristics

	**n (%)**	**Mean**	**SD**
Sex			
Male	10 (83.33)		
Female	2 (16.67)		
Age		68.44	5.08
> 60	-		
60–70	10 (83.33)		
≤ 70	2 (16.67)		
Cognitive age		59.17	6.24
> 60	4 (33.33)		
60–70	7 (58.33)		
≤ 70	1 (8.33)		

All of the participants, except for one, were living with their family or spouse, and all the participant’s self-conceived health status was average or higher. Also, they all had a bachelor's degree or higher, and about half had a diagnosis of hypertension and diabetes.

The questionnaire was administered, and each item was converted to a five-point scale. Wearable device app literacy, which was measured on a scale of 9, was changed to a full scale of 5 using a proportional formula.

**Fig. 4:**
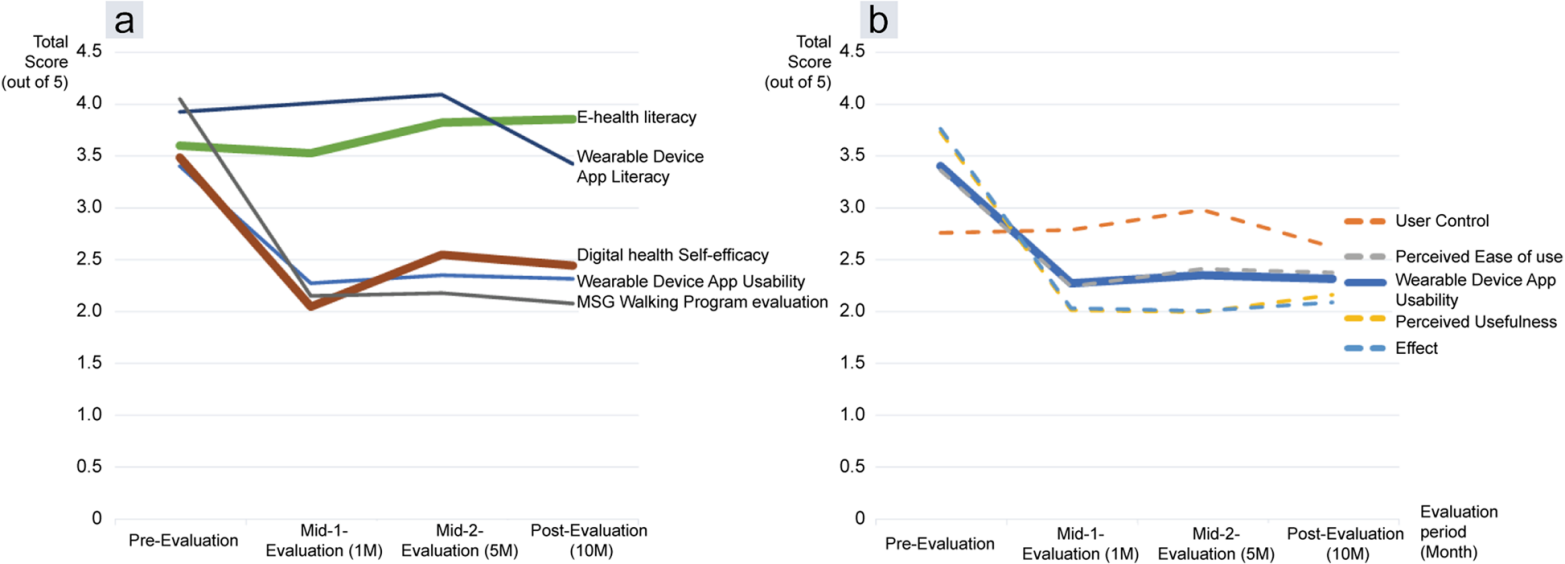
(**a**) E-health literacy, wearable device app literacy, digital health self-efficacy, and MSG walking program evaluation / (**b**) wearable device app usability

The results of the questionnaire are shown in Table 3 and the trends of measured values across time are shown through graphs in Fig. 4.

**Table 3:** Changes in study variables through the MGS walking program

	**Pre-intervention evaluation** **0 w**	**Mid 1-** **Evaluation** **6 w** **(1.4 m)**	**Mid 2-** **Evaluation** **22 w** **(5.1 m)**	**Post-intervention evaluation** **45 w** **(10.3 m)**	**Pre-Post**^**a**^
**Variables**	Mean (SD)	Mean (SD)	Mean (SD)	Mean (SD)	*p*-value (Z)
E-Health Literacy	3.59(0.61)	3.52(0.67)	3.81(0.57)	3.84(0.53)	0.084(-1.730^b^)
Wearable Device App Literacy	7.83(2.41)	8(2)	8.17(2.48)	6.83(3.39)	0.726(-.351^c^)
C**onverted scores** (**1–5**)	4.35(1.34)	4.44(1.11)	4.54(1.38)	3.80(1.88)	
Digital health self-efficacy	3.48(0.72)	2.04(0.55)	2.54(0.61)	2.44(0.52)	0.009^*^(-2.601^**c**^**)**
Wearable Device App Usability	3.39(0.64)	2.26(0.68)	2.34(0.52)	2.3(0.69)	0.023(-2.275^c^)
Effect	3.75(0.61)	2.03(0.6)	2(0.56)	2.08(0.82)	0.007*(-2.706^**c**^**)**
User Control	2.75(0.68)	2.78(0.8)	2.97(0.82)	2.61(0.61)	0.683(-.409^c^)
Perceived Ease of Use	3.35(0.85)	2.23(0.97)	2.4(0.56)	2.37(0.8)	0.062(-1.869^c^)
Perceived Usefulness	3.72(0.9)	2.01(0.67)	1.99(0.63)	2.16(0.9)	0.018^*^(-2.357^**c**^**)**
Program evaluation	4.04(0.58)	2.15(0.59)	2.18(0.7)	2.07(0.64)	0.002^*^(-3.061^**c**^**)**

^a^Wilcoxon signed-rank test

^b^Based on negative rank

^c^Based on the positive rank

*p < 0.05

To check the changes in trends in the study variables, the Wearable Device App Literacy results were converted to scores ranging from 1–5. It was downscaled by multiplying five-ninth (For example, the pre-result value was 7.83, but it was downscaled and expressed as 4.35 points).

The usability of the Mi Fit app measured using the Wearable Device App Usability scale had an initial average score of 3.39 (0.64) out of 5 as shown in Fig. 4(a), which reduced to 2.3 (0.69) in the post-intervention evaluation. Smartphone self-efficacy decreased from 3.48 (0.72) to 2.44 (0.52), and the MSG program evaluation also decreased from 4.04 (0.58) to 2.07 (0.64) across pre- to post-intervention evaluation.

It was observed that the weight of the participants did not change significantly from a mean of 66.13 (7.35) kg to 66.08 (7.04) kg.

In contrast to the score of the app literacy quiz for evaluating the appropriate use of the application, which reduced from 3.92 (1.20) to 3.42 (1.69), the score for e-health literacy increased from 3.59 (0.61) to 3.84 (0.53).

A significant difference was found in smartphone self-efficacy (*p* = 0.009) and the perceived usefulness (*p* = 0.018) dimension of app usability.

The average cognitive age of the participants in the post-intervention evaluation was 59.17 years (6.24), which was 9.27 years lower than the participants' actual mean age, 68.44 years (5.08). Although the effect of factors other than intervention cannot be excluded, the difference between the actual age and the cognitive age was observed to be lower than the actual age of all participants from 3 years old to the largest 20 years old.

The results of the FGI performed 2 months after the intervention were as follows. When asked about the decrease in satisfaction with the program over time, the participants expressed overall satisfaction. They said that this program made them move and feel social connection during COVID-19, however over time, the novelty of the program started diminishing. They reported feeling a sense of monotony in simply measuring the number of steps and answering a series of difficult questions, and a decrease in competitive spirit due to the decrease in participants over time.

When asked about the effect of community activities using social networks on walking exercise programs, participants reported the experience of Hawthorne effect. The Hawthorne effect is a type of reactivity in which individuals modify an aspect of their behavior in response to their awareness of being observed. The participants also stated that the walking course shared by them had a positive effect on walking exercise motivation. Some said that with the new “COVID-19 era” they expected to see the next level of MSG walking program that converges online and offline participation.

Lastly, when asked about their regrets during the walking exercise program and improvements they would want in the future, many participants stated about the inconvenience of the step count verification process. They mentioned that the process of capturing and uploading application screenshots had to be performed manually every day, hence, it would be helpful if it was automatically linked. Additionally, the program was reset at midnight and the records were deleted, which was inconvenient. Participants also complained about the disadvantages of the program with limited face-to-face activities and the need for incentives according to the degree of participation and achievement.

## Discussion

Most existing studies on walking exercise programs for older adults reported changes in their physical and mental functions as a result of the program. However, unlike previous studies, this study applied an exercise program to retired older adults using wearable devices and social media (MSG program) to identify program-related factors that affect program participation.

As the program progressed, the digital literacy of the participants tended to increase. Conversely, the usability evaluation showed a decline over the course of the study.

The questionnaire results showed that the participants’ weight and health status did not change significantly, similar to the results of existing health management studies on adults using wearable devices [40–42].

There were some interesting findings related to usability. The score of the overall evaluation of usability decreased overtime. The pre-intervention evaluations of usability were high due to novelty such as anticipation of a new device that had not been used before. Subsequent evaluations saw a decline because of a change in opinion about the device after actually using it. This is consistent with the results of previous studies [43].

This suggests that the results of long-term use of new devices are more meaningful than the results of initial or one-time use and how the evaluation of new devices can be relatively positive due to the novelty effect.

In the case of the user control dimension of usability evaluation, the results increased from 2.75 (0.68) points in the pre-intervention evaluation to 2.75 (0.68) and 2.97 (0.82) points in the Mid 1 (3 months) and Mid 2-evaluations (5 month), respectively. However, in the post-intervention evaluation the average was at its lowest at 2.61 (0.61) points.

This was expected because at the first evaluation the user is not familiar with the device, and the understanding increases with the increasing usage period. This might also be the result of higher standards of evaluation as knowledge in the relevant field increases.

As a result of indirect confirmation through user control, there was no significant difference between the pre and post results. However, the 'User Control' value rises up to 5 months and then falls, as shown in Fig. 4(b). We estimate that it took approximately five months for the participants to become accustomed to the devices. Of course, it is necessary to take into account the fact that the participants were highly educated and their speed of learning was fast [44, 45].

In the case of e-health literacy, a gradual increase was observed. We believe the long-term use of this application can lead to the overall increase in literacy in the field.

App literacy showed a sharp decline in post-intervention evaluation. Instead of reflecting an actual decrease in participants' app literacy, this is speculated to be a result of the increase in fatigue due to the repeated quiz-type evaluation and decrease in the fidelity of evaluation at a time point 5 months after the last evaluation. Participants may have been annoyed about being exposed to questions about simple app usage that were repeated every day for five months. It might also be the result of insincere participation in the quiz and overconfidence.

Contrary to the researchers’ expectations, participants rated “course sharing” as a useful feature. Initially, the researchers had not thought about course sharing. However, the participants started sharing their own trails by taking pictures and posting them.

Social media was organized in the program as a device to encourage effective program execution and continued use intention of the participant in the Corona situation. However, the participants showed a tendency to enjoy social media activity itself considerably. Clearly, there were previous studies that social media had a positive effect on the health and vitality of the elderly, unlike the dysfunction of social media in relatively young adults and adolescents [46, 47]. However, the participant’s praise for the positive functioning of social media was an unexpected reaction.

In case of the first pre-intervention evaluation, most participants faced difficulties as they had no experience using a mobile questionnaire. The process became easier for the participants from the second questionnaire, which was conducted three months later. This made it possible to indirectly confirm that the ability to navigate the online questionnaire increased while participating in the program. In the evaluations conducted in the 3rd and 5th months, the missing values that were judged to have been a result of participants' inexperience in using the questionnaire were found to be completed. Through this, it was observed that the digital literacy of users increased indirectly. This can be interpreted because participation in programs using information and communication technology, such as this program, increased related competency and digital literacy of older adults [48].

Considering these contents, active use of wearable devices should be considered in the overall health care of the elderly.

In addition, this study was completely conducted online due to COVID-19, but surprisingly, high-quality research and smooth progress were possible. In a situation where social communication was restricted due to social distancing, travel restrictions were applied, and people were mostly staying indoors, this program involving people of similar age and experiences (retirement), getting to share their outdoor activity (outdoor walking/golf) experience, was positively evaluated by participants.

If similar studies are conducted after the pandemic, it will be possible to indirectly measure the effect of social distancing and restrictions caused by the pandemic on online activities.

### Limitations

Although this study has important implications, the statistical results are not normally distributed due to the small sample size. There was no control group to compare the quantitative results of intervention and it was difficult to accurately measure the individual cognitive differences with data collected by self-report. Due to these limitations, the results are difficult to generalize to other older adults. Generalizability is also limited by the fact that the participants of this study were retired older adults from a university in Korea and most participants were highly educated. Also, the cognitive age before the experiment was not measured, so there is a limitation in the absolute evaluation.

However, despite these limitations, the study has significance in that it provided comprehensive analysis by exploring the research from various angles and at various levels using not only quantitative data, but also qualitative data such as observation of program progress and the participant interview and analysis of social network activity.

Using the experience of this study, a follow-up study is needed to collect data in a time series with a control group in a large sample group and to characterize the variables affecting the persistence factor through RM-ANOVA analysis.

## Conclusions

This study analyzed the experiences of retired older adults participating in a walking program using wearable devices and social networks. This autonomous walking exercise program might have had a positive effect on the activities and health level of participants.

Moreover, in the interview, participants emphasized the application of interpersonal strategies and the importance of creating supportive interventions at a social level to promote walking in specific groups.

Although the results of this study cannot be generalized, they can help in identifying appropriate strategies and considerations when planning various exercise or physical activity programs for older adults.

## Data Availability

The data that support the findings of this study are not publicly available due to privacy and ethical concerns, but can be provided upon request to the corresponding author.

## Abbreviations

COVID-19: Coronavirus disease 2019
MSG: MHealth devices and Social media apps using Gamified walking program
STEP: Space, task, equipment, and people
FGI: Focus group interview
